# Shelter dogs and cats as a one health interface for phenotypic and genomic surveillance of antimicrobial-resistant *Enterococcus* Spp. in the United Arab Emirates

**DOI:** 10.3389/fvets.2026.1906971

**Published:** 2026-07-21

**Authors:** Ihab Habib, Khaja Mohteshamuddin, Mohamed-Yousif Ibrahim Mohamed, Glindya Bhagya Lakshmi, Asha Antony, Mohammed Elbediwi

**Affiliations:** 1 Veterinary Public Health Research Laboratory, Department of Veterinary Medicine, College of Agriculture and Veterinary Medicine, United Arab Emirates University, Al Ain, United Arab Emirates; 2 ASPIRE Research Institute for Food Security in the Drylands (ARIFSID), United Arab Emirates University, Al Ain, United Arab Emirates; 3 Veterinary Control Section, Animal Welfare Department, Municipal Services Sector, Abu Dhabi City Municipality, Abu Dhabi, United Arab Emirates; 4 University Institute for Medical Microbiology and Virology, Carl von Ossietzky University Oldenburg, Oldenburg, Germany; 5 Animal Health Research Institute, Agriculture Research Centre, Cairo, Egypt

**Keywords:** antimicrobial resistance, companion animals, *Enterococcus faecalis*, *Enterococcus faecium*, whole-genome sequencing

## Abstract

**Background:**

Companion animal shelters represent underexplored settings for One Health antimicrobial resistance (AMR) surveillance. This study investigated the recovery, antimicrobial resistance, and genomic features of *Enterococcus* spp. from shelter dogs and cats in the United Arab Emirates (UAE).

**Methods:**

Rectal swabs were collected from 230 shelter animals, including 129 dogs and 101 cats, across five shelters in four UAE emirates/cities. *Enterococcus* isolates were recovered using selective culture and identified by MALDI-TOF MS. Confirmed *Enterococcus faecalis* and *Enterococcus faecium* isolates underwent antimicrobial susceptibility testing using Vitek-2. Multidrug-resistant (MDR) and linezolid-resistant isolates were subjected to whole-genome sequencing (WGS) for multilocus sequence typing, resistome analysis, single-nucleotide polymorphism-based phylogeny, phenotype–genotype concordance assessment, and virulence-associated gene profiling.

**Results:**

*Enterococcus* spp. were recovered from 122/230 animals (53.0%; 95% confidence interval: 46.6–59.4), with higher recovery in dogs (71.3% (92/129)) than cats (29.7% (30/101)). Overall, *E. faecalis* accounted for 63/122 isolates (51.6%) and *E. faecium* for 59/122 isolates (48.4%). Phenotypic resistance was most frequent for tetracycline (51.6%), erythromycin (30.3%), ciprofloxacin (27.2%), high-level streptomycin (20.5%), and high-level gentamicin (9.8%). All isolates were susceptible to ampicillin, vancomycin, teicoplanin, and tigecycline. MDR was detected in 25/122 isolates (20.5%) and was more frequent among *E. faecalis* than *E. faecium*. Linezolid resistance was detected in two isolates recovered from two dogs, one male and one female, housed in the same shelter. WGS of 25 MDR or linezolid-resistant isolates revealed diverse sequence types, including dominant *E. faecalis* ST16, shelter-associated SNP clusters, and multiple resistance determinants, including *erm(B), tet(L), tet(M)*, aminoglycoside resistance genes, quinolone resistance-associated mutations, and plasmid-predicted *optrA* in three *E. faecalis* isolates. Virulence-associated genes were more prominent in *E. faecalis*, particularly the endocarditis- and biofilm-associated pilus genes involved in adhesion and biofilm formation, together with a putative exoenzyme-associated factor potentially contributing to host interaction and bacterial fitness.

**Conclusions:**

Shelter dogs and cats in the UAE carried *E. faecalis* and *E. faecium* with phenotypic AMR, MDR profiles, and diverse genomic resistance determinants. The detection of linezolid resistance and plasmid-predicted *optrA* supports inclusion of shelter companion animals in regional One Health AMR surveillance.

## Introduction

Antimicrobial resistance (AMR) is a major One Health challenge that links human, animal, and environmental health through shared antimicrobial use, bacterial populations, and mobile genetic elements (1). Within this context, companion animals have gained increasing attention as potential reservoirs and sentinels of resistant bacteria because of their close contact with humans, frequent exposure to veterinary care, and shared household or community environments (2, 3). Dogs and cats may carry antimicrobial-resistant bacteria asymptomatically, including organisms of clinical relevance, and may contribute to the circulation of resistance determinants across human–animal interfaces (2, 3). This role is particularly relevant in high-contact settings such as shelters, where animal stress, density and turnover, along with previous antimicrobial exposure and variable health status may favor bacterial transmission and persistence (4).

*Enterococcus* spp. are commensal members of the gastrointestinal microbiota of humans and animals, but they are also opportunistic pathogens capable of causing infections in both human and veterinary settings (5). Their ability to survive under diverse environmental conditions, acquire resistance determinants, and exchange genetic material with other bacteria makes them important indicators for AMR surveillance (5). Enterococci, particularly *Enterococcus faecalis* and *Enterococcus faecium*, have been widely used to monitor antimicrobial selection pressure and the dissemination of resistance genes in animals, humans, food, and the environment (6). Previous studies in companion animals have shown that enterococci from dogs and cats may carry resistance to multiple antimicrobial classes, including macrolides, tetracyclines, aminoglycosides, and other agents used in human and veterinary medicine, supporting their relevance as reservoirs of resistance genes with potential One Health implications (5, 6).

Of particular concern is the emergence of resistance to critically important antimicrobials used for the treatment of serious Gram-positive infections (7, 8). Vancomycin-resistant enterococci, especially vancomycin-resistant *E. faecium*, are recognized as important healthcare-associated pathogens because therapeutic options are limited (7, 8). Linezolid is commonly regarded as an important alternative for infections caused by multidrug-resistant Gram-positive bacteria, including vancomycin-resistant enterococci (9). Although linezolid resistance in enterococci remains relatively uncommon compared with resistance to older antimicrobial classes, its detection is concerning because resistance can be mediated by transferable determinants, including *cfr, optrA*, and *poxtA*, or by chromosomal mutations (9, 10). The occurrence of such resistance in animal-associated enterococci, even when detected incidentally, warrants careful characterization because it may indicate hidden reservoirs of clinically relevant resistance mechanisms outside hospital settings (10).

In the United Arab Emirates (UAE), shelter dogs and cats represent an underexplored population within One Health AMR surveillance. Despite the growing recognition of companion animals as reservoirs and sentinels of antimicrobial-resistant bacteria (11, 12), limited data are available on the species distribution, antimicrobial resistance profiles, and genomic features of *Enterococcus* spp. circulating in the understudied shelter environments in the UAE. This knowledge gap may represent a blind spot in national and regional AMR surveillance, particularly because shelters can receive animals with diverse backgrounds and unknown antimicrobial exposure history (4). Therefore, this study aimed to isolate and identify *Enterococcus* spp. from shelter dogs and cats in the UAE, characterize their phenotypic antimicrobial resistance profiles, determine the occurrence of multidrug resistance (MDR), and use whole-genome sequencing (WGS) to investigate the genomic features and resistance determinants of MDR isolates. To our knowledge, this study represents one of the first phenotypic and genomic investigations of *Enterococcus* spp. from shelter companion animals in the UAE and the wider Middle East, addressing an important gap in One Health AMR surveillance and documents the incidental detection of linezolid resistance, underscoring the need to include shelter animals in regional AMR monitoring frameworks.

## Materials and methods

### Sampling of shelter dogs and cats

The study design was intended to provide baseline data on *Enterococcus* spp. carriage and AMR among shelter dogs and cats in the UAE. Shelter participation was based on a voluntary approach, whereby shelter management teams that were willing to collaborate and provide access to animals and relevant metadata were enrolled. This targeted recruitment strategy was considered appropriate for the exploratory nature of the study (13), as it allowed the inclusion of animals from multiple geographic locations, while ensuring feasibility, animal welfare, and cooperation with shelter personnel. A survey was conducted among shelter dogs and cats in the UAE from April to June 2025. The study population comprised companion animals housed in five animal shelters located across four UAE emirates/cities: Abu Dhabi, Ajman, Umm Al Quwain, and Fujairah. A total of 230 animals were sampled, including 129 dogs and 101 cats. The number of animals sampled per shelter was determined by animal availability, shelter capacity, and accessibility during sampling. The studied population included a convenience sample of 39 animals from shelter A in Abu Dhabi, 60 from shelter B in Umm Al Quwain, 30 from shelter C in Ajman, 51 from shelter D in Fujairah, and 50 from shelter E in Ajman. Shelter A included both dogs and cats, shelters B and D included dogs only, and shelters C and E included cats only.

The study was covered by Animal Ethics certificate No. ERA_2024_5349, “titled: use of animals for research and students training on non-invasive procedures”, reviewed and approved by United Arab Emirates University Animal Ethics Committee. No invasive procedures were performed as part of this study. Rectal swabs were collected from each animal using sterile plastic shafted swabs by gently inserting the swab into the rectum and rotating it to obtain fecal material (12). Each swab was immediately placed into Cary-Blair transport medium (HiMedia Laboratories, India) (12), labeled with a unique animal identifier linked to the corresponding metadata sheet, maintained under refrigerated conditions, and transported to the laboratory for microbiological processing as soon as possible.

### Isolation and species-level identification of *Enterococcus* spp.

Rectal swabs collected from shelter dogs and cats were processed for the isolation of *Enterococcus* spp. upon arrival at the laboratory. Each swab was homogenized in 10 ml buffered peptone water, and an aliquot (100 μl) of the suspension was streaked onto Slanetz–Bartley agar for selective isolation of enterococci (14). Plates were incubated aerobically at 41.5 °C for 36–48 h, and colonies showing typical enterococcal morphology were selected for further characterization (14). Presumptive *Enterococcus* isolates were purified by subculture on Nutrient Agar and subjected to species-level identification using matrix-assisted laser desorption ionization–time-of-flight mass spectrometry (MALDI-TOF MS) with the Autobio ms1000 system (Autobio Diagnostics, Zhengzhou, China) (14). Confirmed isolates were stored at −80 °C in bead-based cryopreservation tubes (Microbank™, Pro-Lab Diagnostics, Canada) until antimicrobial susceptibility testing and subsequent molecular characterization.

### Phenotypic antimicrobial susceptibility testing of *E. faecalis* and *E. faecium*

Confirmed *Enterococcus faecalis* and *Enterococcus faecium* isolates were selected for phenotypic antimicrobial susceptibility testing because of their recognized clinical and One Health relevance. Antimicrobial susceptibility testing was performed using the Vitek-2 automated system with the AST-P592 card (bioMérieux, France), following the manufacturer's instructions and adopting breakpoints from CLSI (Clinical and Laboratory Standards Institute) (15). The antimicrobial panel included agents relevant to enterococcal infections and AMR surveillance: β-lactams, represented by ampicillin (resistance breakpoint ≥32 μg/ml); macrolides, represented by erythromycin (resistance breakpoint ≥8 μg/ml); fluoroquinolones, represented by ciprofloxacin (resistance breakpoint ≥8 μg/ml); aminoglycosides, represented by high-level gentamicin (resistance breakpoint ≥500 μg/ml) and high-level streptomycin (resistance breakpoint ≥1000 μg/ml); tetracyclines, represented by tetracycline (resistance breakpoint ≥16 μg/ml); glycopeptides, represented by vancomycin (resistance breakpoint ≥32 μg/ml) and teicoplanin (resistance breakpoint ≥32 μg/ml); glycylcyclines, represented by tigecycline (resistance breakpoint ≥2 μg/ml); and oxazolidinones, represented by linezolid (resistance breakpoint ≥8 μg/ml). Quality control for antimicrobial susceptibility testing was performed using *Enterococcus faecalis* ATCC 29212 as the reference strain (15). Isolates showing resistance to at least one agent in three or more antimicrobial classes were classified as MDR. Isolates with MDR phenotypes, or with unusual resistance to critically important antimicrobials (e.g. linezolid) were prioritized for WGS.

### WGS and bioinformatic characterization of MDR *Enterococcus* isolates

Genomic DNA was extracted from overnight pure cultures using the Wizard^®^ Genomic DNA Purification Kit (Promega, Madison, WI, USA), according to the manufacturer's instructions. The concentration and purity of the extracted DNA were assessed prior to library preparation using spectrophotometric and fluorometric quality-control measurements (NanoDrop™ spectrophotometer, Thermo Fisher Scientific, USA; Qubit™ fluorometer, Thermo Fisher Scientific, USA). Library preparation and sequencing were performed by the service provider Novogene (Cambridge, UK) using the NovaSeq short-read sequencing platform (Illumina, San Diego, CA, USA).

Short reads were quality controlled and trimmed using fastp v0.23.4 (https://github.com/OpenGene/fastp) and assembled using Shovill v1.1.0 (https://github.com/tseemann/shovill). Genome assemblies were subsequently analyzed using the Solu platform v1.0.702 (Solu Healthcare Inc., Helsinki, Finland), which enables standardized batch processing and genomic characterization of bacterial isolates (16). The Solu workflow was used for *in silico* species identification using the Kraken2 taxonomic classification (https://github.com/DerrickWood/kraken2). Solu pipeline identified multilocus sequence types (MLST) using mlst v2.32.2 (https://github.com/tseemann/mlst). The detection of antimicrobial resistance genes and resistance-associated chromosomal mutations was annotated with AMRFinderPlus v4.0.23-2025-07-16.1 (https://github.com/ncbi/amr), using the “organism” parameter for species-specific point mutations. Screening for putative virulence-associated determinants was performed using ABRicate v1.0.1 (https://github.com/tseemann/abricate) against the Virulence Factor Database (VFDB) (https://www.mgc.ac.cn/VFs/). Antimicrobial resistance and virulence gene detection were performed using predefined sequence-similarity thresholds, where genes were considered present when they met a minimum sequence identity of ≥95% and a minimum sequence coverage of ≥60% (16).

Finally, SNP-based phylogeny was inferred to align same-species samples (reference-based alignment) and compare them at single-nucleotide resolution. Samples were grouped into clusters using a 20-SNP single-linkage clustering threshold (17). A phylogenetic tree for each species is inferred with the Solu embedded IQ-TREE v2.3.6 (http://www.iqtree.org) maximum-likelihood algorithm (16).

## Results

### Recovery and species distribution of *Enterococcus* spp. from shelter dogs and cats

A total of 230 shelter animals were sampled, comprising 129 dogs and 101 cats from five shelters across four emirates/cities in the UAE. Overall, *Enterococcus* spp. were recovered from 122 animals, corresponding to a recovery rate of 53.0% (122/230; 95% confidence interval (CI): 46.6–59.4) (Table 1). Recovery was higher in dogs than in cats, with *Enterococcus*-positive samples detected in 92/129 dogs (71.3%; 95% CI: 63.0–78.4) and 30/101 cats (29.7%; 95% CI: 21.7–39.2) (Table 1). This difference by host species was statistically significant (Fisher's exact test, *p* < 0.001). Recovery was highest in the two dog-only shelters: Shelter B in Umm Al Quwain, where 52/60 dogs were positive (86.7%), followed by Shelter D in Fujairah, where 36/51 dogs were positive (70.6%) (Table 1).

**Table 1:** Overall recovery, host-level and shelter-level distributions of *Enterococcus* spp. recovered from shelter dogs and cats in the United Arab Emirates.

Category	Shelter/Emirate	Animal species	Total animals sampled, *N*	*Enterococcus*-positive samples, *n/N* (%; 95% CI)^*^	*E. faecalis*, *n* (% isolates)^**^	*E. faecium*, *n* (% isolates)^**^
Host-level	All subjects	Cat, Dog	230	122/230 (53.0; 46.6-59.4)	63 (51.6)	59 (48.4)
		Dog	129	92/129 (71.3; 63.0-78.4)	39 (42.4)	53 (57.6)
		Cat	101	30/101 (29.7; 21.7-39.2)	24 (80.0)	6 (20.0)
Shelter-level	Shelter A/Abu Dhabi	Cat, Dog	39	11/39 (28.2; 16.5-43.8)	7 (63.6)	4 (36.4)
	Shelter B/Umm Al Quwain	Dog	60	52/60 (86.7; 75.8-93.1)	6 (11.5)	46 (88.5)
	Shelter C/Ajman	Cat	30	4/30 (13.3; 5.3-29.7)	3 (75.0)	1 (25.0)
	Shelter D/Fujairah	Dog	51	36/51 (70.6; 57.0-81.3)	31 (86.1)	5 (13.9)
	Shelter E/Ajman	Cat	50	19/50 (38.0; 25.9-51.8)	16 (84.2)	3 (15.8)

^*^Percentages for Enterococcus-positive samples were calculated using the total number (N) of animals sampled in each category as the denominator. The 95% confidence intervals (CI) were calculated using the Wilson method; ^**^Species percentages were calculated using the number of Enterococcus-positive samples/confirmed isolates in the corresponding row as the denominator.

Among the 122 confirmed isolates, *E. faecalis* accounted for 63 isolates (51.6%), while *E. faecium* accounted for 59 isolates (48.4%) (Table 1). Species distribution differed by host species (Fisher's exact test, *p* < 0.001). In dogs, *E. faecium* was more frequent than *E. faecalis* (53/92, 57.6 vs. 39/92, 42.4%), whereas in cats, *E. faecalis* predominated (24/30, 80.0%) over *E. faecium* (6/30, 20.0%). At the shelter level, *E. faecium* predominated in Shelter B (46/52, 88.5%), while *E. faecalis* was the predominant species in Shelter A (7/11, 63.6%), Shelter C (3/4, 75.0%), Shelter D (31/36, 86.1%), and Shelter E (16/19, 84.2%) (Table 1).

### Phenotypic antimicrobial resistance profiles of *Enterococcus* isolates

Antimicrobial susceptibility testing was performed on all of the 122 confirmed *Enterococcus* isolates (Table 2). Ciprofloxacin results were available/interpretable for 114 isolates only; the remaining 8 isolates had missing or non-reportable ciprofloxacin susceptibility results, so they were excluded from the denominator for that antimicrobial. All isolates were susceptible to ampicillin, teicoplanin, vancomycin, and tigecycline. Resistance was most frequently detected against tetracycline, with 63/122 isolates classified as resistant (51.6%), followed by erythromycin (37/122, 30.3%), ciprofloxacin (31/114, 27.2%), high-level streptomycin (25/122, 20.5%), and high-level gentamicin (12/122, 9.8%) (Table 2). Linezolid resistance was phenotypically detected in two isolates (2/122, 1.6%), both from Shelter D, while two additional isolates from Shelter E showed intermediate susceptibility to linezolid (Table 2). Overall, 25/122 isolates (20.5%) were classified as MDR, with higher MDR among *E. faecalis* isolates than *E. faecium* isolates (18/63, 28.6 vs. 7/59, 11.9%; Fisher's exact test, *p* = 0.026). At the host level, multidrug resistance was detected in 14/30 cat isolates (46.7%) and 11/92 dog isolates (12.0%) (Fisher's exact test, *p* < 0.001).

**Table 2:** Phenotypic antimicrobial resistance patterns of *Enterococcus* spp. isolates recovered from shelter dogs and cats in the United Arab Emirates.

Antimicrobial	Resistance status^*^	Overall (*n* = 122)	Shelter A (*n* = 11)	Shelter B (*n* = 52)	Shelter C (*n* = 4)	Shelter D (*n* = 36)	Shelter E (*n* = 19)
Ampicillin	S	122 (100)	11 (100)	52 (100)	4 (100)	36 (100)	19 (100)
Gentamicin, high-level	R	12 (9.8)	4 (36.4)	1 (1.9)	3 (75)	1 (2.8)	3 (15.8)
	S	110 (90.2)	7 (63.6)	51 (98.1)	1 (25)	35 (97.2)	16 (84.2)
Streptomycin, high-level	R	25 (20.5)	5 (45.5)	6 (11.5)	1 (25)	6 (16.7)	7 (36.8)
	S	97 (79.5)	6 (54.5)	46 (88.5)	3 (75)	30 (83.3)	12 (63.2)
Ciprofloxacin	R	31 (27.2)	5 (45.5)	17 (38.6)	4 (100)	3 (8.3)	2 (10.5)
	I	29 (25.4)	2 (18.2)	10 (22.7)	0 (0.0)	12 (33.3)	5 (26.3)
	S	54 (47.4)	4 (36.4)	17 (38.6)	0 (0.0)	21 (58.3)	12 (63.2)
Erythromycin	R	37 (30.3)	8 (72.7)	8 (15.4)	4 (100)	6 (16.7)	11 (57.9)
	I	72 (59)	3 (27.3)	42 (80.8)	0 (0.0)	21 (58.3)	6 (31.6)
	S	13 (10.7)	0 (0.0)	2 (3.8)	0 (0.0)	9 (25)	2 (10.5)
Linezolid	R	2 (1.6)	0 (0.0)	0 (0.0)	0 (0.0)	2 (5.6)	0 (0.0)
	I	2 (1.6)	0 (0.0)	0 (0.0)	0 (0.0)	0 (0.0)	2 (10.5)
	S	118 (96.7)	11 (100)	52 (100)	4 (100)	34 (94.4)	17 (89.5)
Teicoplanin	S	122 (100)	11 (100)	52 (100)	4 (100)	36 (100)	19 (100)
Vancomycin	S	122 (100)	11 (100)	52 (100)	4 (100)	36 (100)	19 (100)
Tetracycline	R	63 (51.6)	8 (72.7)	26 (50)	4 (100)	12 (33.3)	13 (68.4)
	I	1 (0.8)	0 (0.0)	0 (0.0)	0 (0.0)	0 (0.0)	1 (5.3)
	S	58 (47.5)	3 (27.3)	26 (50)	0 (0.0)	24 (66.7)	5 (26.3)
Tigecycline	S	122 (100)	11 (100)	52 (100)	4 (100)	36 (100)	19 (100)

^*^R, resistant; I, intermediate; S, susceptible.

**Figure 1:**
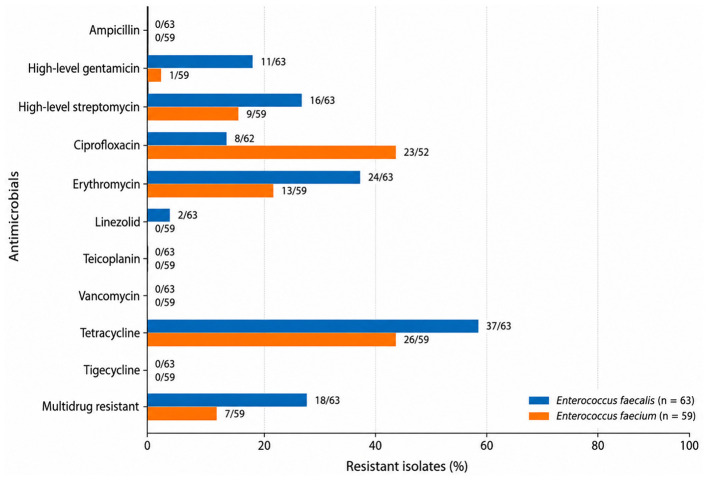
Species-specific phenotypic antimicrobial resistance among *Enterococcus faecalis* and *Enterococcus faecium* from shelter dogs and cats in the United Arab Emirates.

Species-specific resistance patterns are shown in Figure 1. Resistance to high-level gentamicin was more frequent in *E. faecalis* than in *E. faecium* (11/63, 17.5 vs. 1/59, 1.7%; Fisher's exact test, *p* = 0.004). In contrast, ciprofloxacin resistance was more frequent in *E. faecium* than in *E. faecalis* (23/52, 44.2 vs. 8/62, 12.9%; Fisher's exact test, *p* < 0.001). The two phenotypically linezolid-resistant isolates belonged to *E. faecalis* (Figure 1).

### Genomic diversity and population structure of MDR isolates

**Figure 2:**
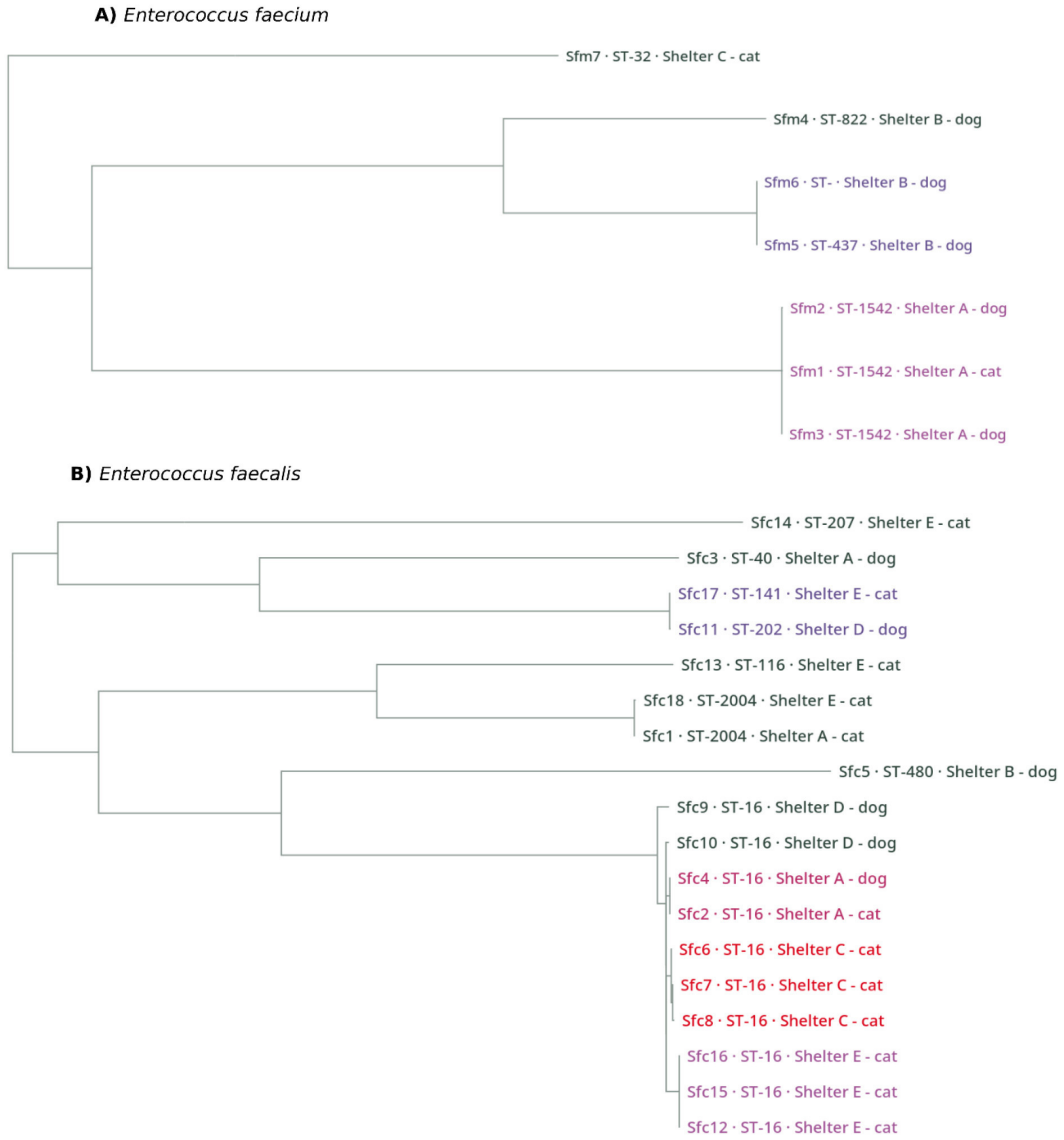
**(A, B)** Maximum-likelihood single-nucleotide (SNP) polymorphism-based phylogenies of *Enterococcus faecium* and *Enterococcus faecalis* isolates from shelter dogs and cats in the United Arab Emirates, annotated with sequence type (ST), shelter (coded letters A to E), and host species (cat/dog) metadata and grouped (colored clusters) using a 20 SNP single-linkage threshold.

In relation to their phenotypic resistance profile, WGS was performed on 25 MDR and linezolid-resistant isolates, comprising seven *E. faecium* and 18 *E. faecalis* isolates (Figure 2). Four sequence types (STs) were assigned among the seven *Enterococcus faecium* isolates. ST1542 was the most frequent sequence type and included three isolates, Sfm1, Sfm2, and Sfm3, all recovered from Shelter A. In SNP-based phylogeny (Figure 2), the three ST1542 isolates from Shelter A grouped together within the same 20-single-nucleotide polymorphism cluster. Among the 18 *E. faecalis* isolates, ST16 was the dominant sequence type, accounting for 10 isolates. These ST16 isolates were recovered from multiple shelters, including Shelter A, Shelter C, Shelter D, and Shelter E (Figure 2). Several ST16 isolates grouped into 20-SNP clusters with shared shelter metadata, including a cluster comprising Sfc6, Sfc7, and Sfc8 from Shelter C, and another comprising Sfc12, Sfc15, and Sfc16 from Shelter E. A smaller ST16 cluster included Sfc2 and Sfc4 from Shelter A (Figure 2).

### WGS-based resistome profiles

**Figure 3:**
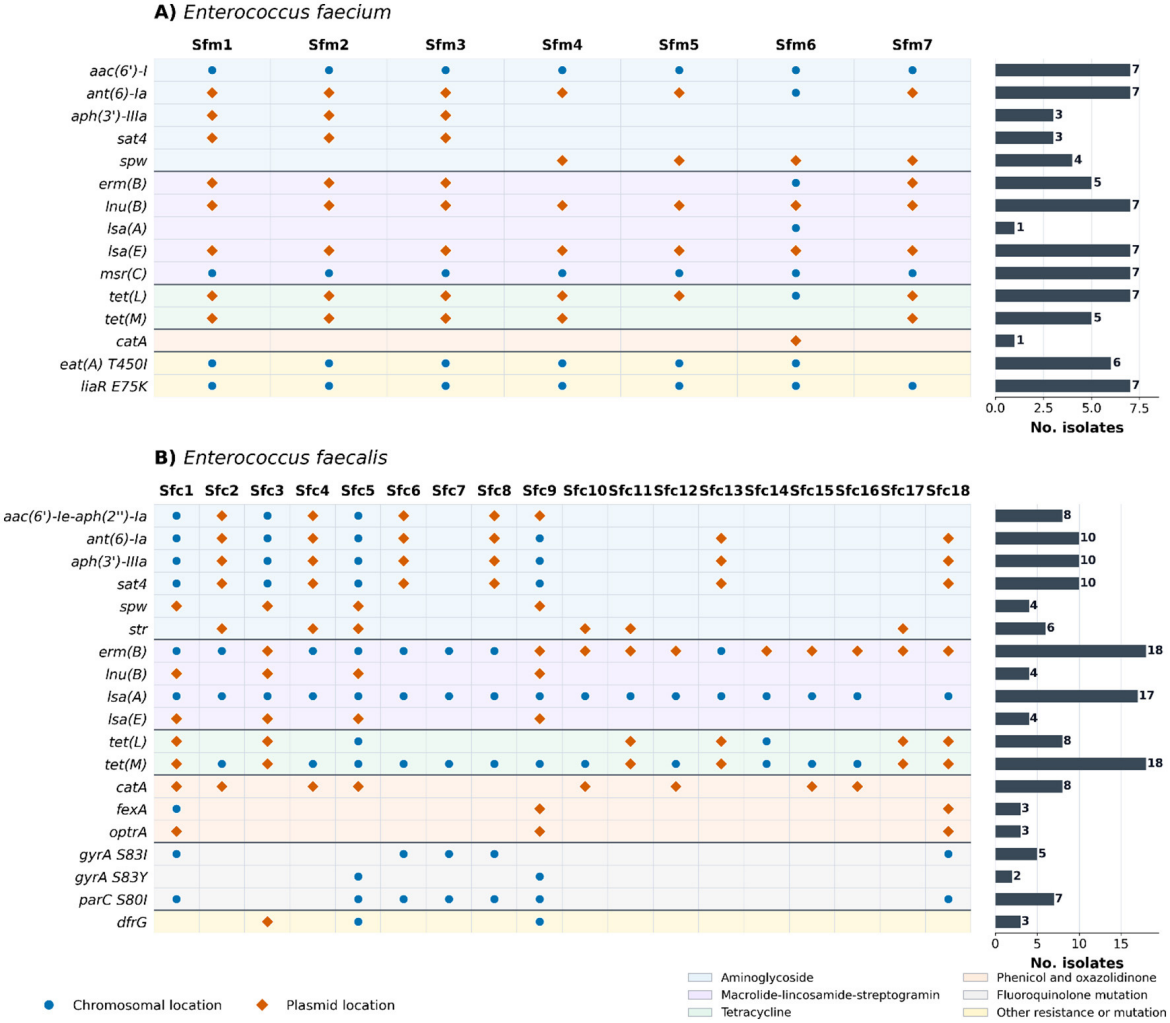
**(A, B)** Whole-genome sequencing based resistome profiles and predicted locations of antimicrobial resistance determinants (listed on vertical axis) in multidrug-resistant *Enterococcus faecium* (*n* = 7) and *Enterococcus faecalis* (*n* = 18) isolates (codes of isolates are listed on top of each panel) recovered from shelter dogs and cats in the United Arab Emirates.

WGS identified diverse AMR determinants among the 25 MDR isolates (Figure 3). Among the seven *E. faecium* isolates, aminoglycoside-associated determinants included *aac(6*′*)-I* (aminoglycoside acetyltransferase) and *ant(6)-Ia* (aminoglycoside nucleotidyltransferase), both were detected in all isolates. Additional aminoglycoside-associated genes included *aph(3*′*)-IIIa* (aminoglycoside phosphotransferase) and *sat4* (streptothricin acetyltransferase), each detected in 3/7 isolates, and *spw* (streptomycin resistance), detected in 4/7 isolates. Macrolide-, lincosamide-, and streptogramin-associated determinants included *erm(B)* in 5/7 isolates, while *lnu(B), lsa(E)*, and *msr(C)* were detected in all *E. faecium* isolates. Tetracycline resistance determinants included *tet(L)* in 7/7 isolates and *tet(M)* in 5/7 isolates. The phenicol resistance gene *catA* (chloramphenicol acetyltransferase) was detected in one *E. faecium* isolate. Resistance-associated mutations included *eat(A)* T450I (resistance-associated mutation) in six isolates and *liaR* E75K (cell-envelope stress response-associated mutation) in all seven isolates (Figure 3).

Among the 18 *E. faecalis* isolates, aminoglycoside-associated determinants included *aac(6*′*)-Ie-aph(2*″*)-Ia* in 8/18 isolates, *ant(6)-Ia* in 10/18 isolates, *aph(3*′*)-IIIa* in 10/18 isolates, *sat4* in 10/18 isolates, *spw* in 4/18 isolates, and *str* in 6/18 isolates. The macrolide resistance gene *erm(B)* was detected in all 18 isolates (Figure 3). The key macrolide-, lincosamide-, and streptogramin-associated determinants was *lsa(A)*, detected in 17/18 isolates. Tetracycline resistance determinants were frequent, with *tet(M)* detected in all 18 isolates and *tet(L)* detected in 8/18 isolates. Phenicol-associated genes included *catA* in 8/18 isolates and *fexA* in 3/18 isolates. The oxazolidinone and phenicol resistance gene *optrA* was detected in three *E. faecalis* isolates (Figure 3).

The predicted genomic location of resistance determinants varied by gene and species (Figure 3). In *E. faecium*, several determinants were predicted to be chromosomal, including *aac(6*′*)-I, msr(C), eat(A)* T450I, and *liaR* E75K, while multiple aminoglycoside-, macrolide/lincosamide-, and tetracycline-associated determinants were detected on predicted plasmid-associated contigs in one or more isolates. In *E. faecalis*, resistance determinants were also distributed between predicted chromosomal and plasmid-associated locations.

The three *optrA*-positive *E. faecalis* isolates showed a predicted plasmid location for this determinant (Figure 3). The hypothetical genetic environment for the plasmid bearing *optrA*-positive *E. faecalis* is depicted in Supplementary Figure 1, revealing that *optrA* and *fexA*, together with *araC*, a transcriptional regulator, were co-localized on the same predicted plasmid context in all three *optrA*-positive *E. faecalis* (Sfc1, Sfc9, and Sfc18).

### Phenotype–genotype concordance of antimicrobial resistance

**Figure 4:**
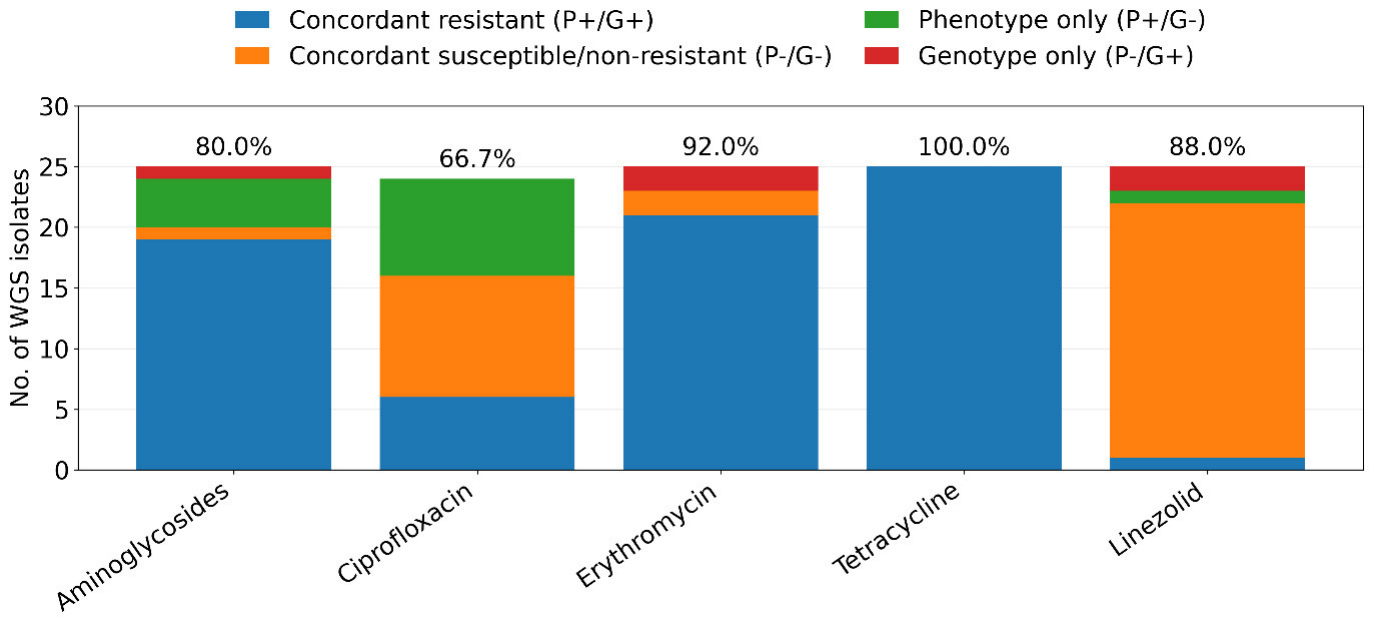
Phenotype–genotype concordance of antimicrobial resistance among 25 multidrug-resistant *Enterococcus isolates* from shelter dogs and cats in the United Arab Emirates. Bars show concordant and discordant resistance classifications based on Vitek-2 phenotypes and whole-genome sequencing-detected resistance determinants. Percentages on top of the bar indicate overall concordance by antimicrobial category. Intermediate phenotypes were treated as non-resistant for the concordance summary.

Phenotype–genotype concordance was assessed across antimicrobial categories among the 25 genome sequenced isolates (Figure 4). Tetracycline showed complete phenotype–genotype concordance; with all 25 isolates were phenotypically tetracycline-resistant and carried *tet(L)* and/or *tet(M)*. Erythromycin showed high concordance, with 21 phenotype-positive/genotype-positive, corresponding to 92.0% overall concordance. For linezolid, the overall concordance was 88.0%, with one phenotype-positive/genotype-positive isolate, 21 phenotype-negative/genotype-negative isolates, one phenotype-positive/genotype-negative isolate, and two phenotype-negative/genotype-positive isolates. The phenotype-positive/genotype-negative linezolid isolate was Sfc10, while genotype-positive/phenotype-negative linezolid profiles were observed in Sfc1 and Sfc18. Ciprofloxacin showed the lowest concordance among the evaluated categories, with an overall concordance of 66.7% (Figure 4).

### Virulence-associated gene profiles of genome-sequenced isolates

**Figure 5:**
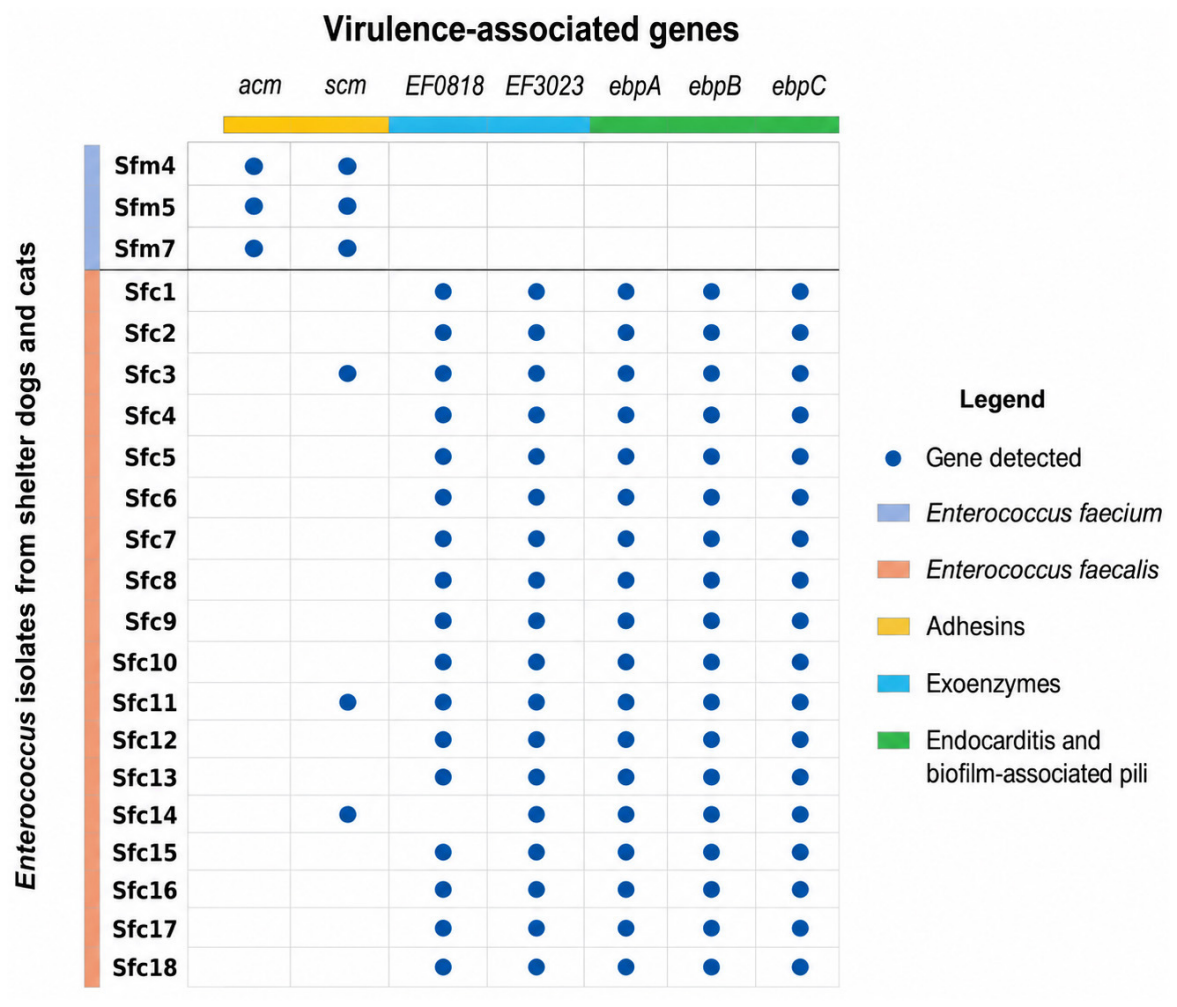
Heatmap showing the distribution of virulence-associated genes among *Enterococcus faecium* and *Enterococcus faecalis* isolates from shelter dogs and cats in the United Arab Emirates, grouped by species and functional gene category.

Among the *E. faecium* isolates, the adhesin-associated genes *acm* (collagen-binding adhesin) and *scm* (surface collagen adhesin) were the only detected genes in three of the seven isolates (Figure 5). The genes *ebpA, ebpB*, and *ebpC* (genes encoding endocarditis- and biofilm-associated pilus components) were detected in all 18 *E. faecalis* isolates. Exoenzyme-associated determinants were also detected, with *EF3023* present in 17/18 isolates and *EF0818* present in 3/18 isolates. The adhesin-associated genes *acm* and *scm* were not detected among the *E. faecalis* isolates shown in Figure 5.

## Discussion

This study provides baseline phenotypic and genomic evidence that shelter dogs and cats in the UAE carry *E. faecalis* and *E. faecium* with diverse antimicrobial resistance phenotypes and genomic determinants. To our knowledge, this is among the first investigations in the UAE and wider Middle East to examine *Enterococcus* spp. from the understudied context of shelter companion animals. This is relevant because enterococci can serve as both commensal bacteria and opportunistic pathogens, and their persistence, genomic plasticity, and capacity to acquire resistance genes make them useful indicators for AMR surveillance across animal, human, and environmental interfaces (6, 18).

The overall recovery pattern in the present study indicated that *Enterococcus* carriage was common among the sampled shelter population, with higher detection in dogs than in cats. This host-associated pattern was also reflected at the shelter level, where the greatest recovery was observed in the two dog-only shelters, suggesting that dog-housing environments in the surveyed shelters might have contributed to the overall recovery profile. However, this interpretation should remain cautious because the study was cross-sectional and not designed to distinguish biological host effects from shelter-level management or environmental influences. From a broader perspective, our findings are consistent with the recognized occurrence of enterococci in the intestinal microbiota of companion animals reported elsewhere. In Northeast China, enterococcal isolation rates was reported as 93.79% from police dogs, 69.90% from pet dogs, and 76.67% from pet cats, suggesting that host type and living environment can influence enterococcal recovery (19). In Italy, Iseppi and colleagues detected enterococci in all 115 healthy dogs and cats sampled (20). Overall, the present UAE findings are consistent with international reports while adding rare regional data from the Middle East. They are also distinctive in focusing on shelter dogs and cats, a population still underrepresented in One Health AMR surveillance (4, 21).

Regarding species level detection, our results show that no other species than *E. faecalis* and *E. faecium* were recovered from the characterized shelter-associated enterococcal population in the UAE. Our data revealed the predominance of *E. faecium* among dog isolates and *E. faecalis* among cat isolates suggests that host-related factors may influence the structure of enterococcal carriage in this shelter setting. This distribution contrasts with an Italian study, where *E. faecium* (36.5%) and *E. faecalis* (31.3%) were the most frequent species but with lower proportions than in the present UAE dataset (20). In Portugal, companion animal isolates from healthy animals were predominantly *E. faecalis* (61%), followed by *E. faecium* (29%) and *E. hirae* (10%) (21). In contrast, clinical isolates from dogs and cats in southern Brazil were dominated by *E. faecium* (68.4%), followed by *E. faecalis* (29.8%) (22). These differences likely reflect the contrast between healthy carriage, shelter populations, and clinical submissions, and support the need to interpret species distribution within the sampling context.

In the present study, phenotypic resistance was concentrated mainly in antimicrobial classes commonly associated with acquired resistance in enterococci, particularly tetracyclines, macrolides, fluoroquinolones, and high-level aminoglycosides. This pattern indicates that the shelter-associated isolates carried resistance profiles relevant to both veterinary and One Health AMR surveillance, while resistance to last-line agents remained uncommon. This profile resembles several companion animal studies in which tetracycline and macrolide resistance are repeatedly prominent. De Leener and coworkers reported tetracycline resistance in 55% of canine and feline enterococci (23), with *tet(M)* and *erm(B)* as major mechanisms like what we observed in the present study. In China, similarly, tetracycline and erythromycin were reported as the predominant resistance phenotypes among enterococci from dogs and cats (19). However, the overall MDR frequency in the present study was lower than that reported in several clinical or higher-risk companion animal cohorts. In the UAE shelter population, 20.5% of the isolates were classified as MDR, compared with 78.9% of the isolates recovered from hospitalized dogs and cats in southern Brazil (22) and 68.92% of enterococci from dogs and cats in Northeast China (19). This difference is expected when comparing commensal shelter carriage with clinical infection isolates, where antimicrobial exposure and disease status may enrich resistant populations.

The complete susceptibility to ampicillin, vancomycin, teicoplanin, and tigecycline in the UAE collection is notable. The absence of vancomycin resistance agrees with a study in Belgium, where vancomycin-resistant enterococci were not detected among pet isolates (23), and a study in Brazil, where none of the 57 isolates showed resistance to vancomycin or linezolid (22). However, vancomycin resistance in companion animal enterococci has been reported elsewhere (24, 25). Together, these reports support the value of continued surveillance even when phenotypic vancomycin resistance is not detected in a given cross-sectional study. From a One Health perspective, because shelter animals may transition into household environments through adoption, even rare resistance phenotypes or emerging determinants could become relevant at the human–companion animal interface.

Linezolid resistance was rare in the UAE collection, detected phenotypically in two isolates, with two additional linezolid-intermediate isolates. Nevertheless, its detection is important because linezolid is a key option for difficult-to-treat Gram-positive infections, including vancomycin-resistant enterococci (9). WGS detected *optrA* in three *E. faecalis* isolates, all predicted to be plasmid-associated, and *fexA* (phenicol resistance) was co-detected in the *optrA*-positive subset, matching with previous finding from *optrA*-positive chicken meat isolated in the UAE and elsewhere (9, 10). Because linezolid is not licensed or routinely used in small-animal practice in the UAE, the detection of *optrA* is unlikely to reflect direct linezolid selection in these animals and may instead indicate co-selection through linked phenicol resistance, exposure to other antimicrobial pressures, or acquisition of mobile resistance elements from broader animal, food, or environmental reservoirs as pointed in other studies (10, 26). The present finding does not establish zoonotic transmission, but it identifies shelter companion animals as a potential surveillance point for emerging linezolid resistance.

The WGS resistome further explained the main phenotypic resistance patterns. In *E. faecium*, the detection of *aac(6*′*)-I, ant(6)-Ia, aph(3*′*)-IIIa, sat4*, and *spw* was consistent with aminoglycoside resistance potential, while *erm(B), lnu(B), lsa(E)*, and *msr(C)* reflected macrolide, lincosamide, and streptogramin resistance determinants. The widespread detection of *tet(L)* and *tet(M)* supported the high tetracycline phenotypic resistance. In *E. faecalis*, the resistome included *aac(6*′*)-Ie-aph(2*″*)-Ia, ant(6)-Ia, aph(3*′*)-IIIa, sat4, str, erm(B), lsa(A), lsa(E), tet(L), tet(M), catA, fexA, optrA, dfrG*, and quinolone resistance-associated substitutions in *gyrA* and *parC*. This diversity is consistent with the capacity of enterococci to acquire antimicrobial resistance genes through mobile genetic elements and chromosomal adaptation (18). The predicted plasmid association of several determinants, particularly *optrA, fexA*, tetracycline, and aminoglycoside resistance genes, is relevant for genomic surveillance, although experimental validation would be required to confirm transferability. Viewed through a One Health lens, such plasmid-associated resistance contexts are important because they may facilitate persistence and exchange of resistance determinants across animal, human, food, and environmental interfaces (26, 27).

Phenotype–genotype concordance analysis indicated that WGS largely explained the main resistance phenotypes, particularly for tetracycline and erythromycin, where resistance aligned closely with detection of *tet(L)*/*tet(M)* and *erm(B)*, respectively. This agrees with earlier companion animal studies in which *tet(M)* and *erm(B)* were major determinants underlying tetracycline and macrolide-lincosamide resistance in pet-associated enterococci (23). By contrast, the lower concordance observed for ciprofloxacin and aminoglycosides, and the discordant linezolid profiles, underscore the complexity of inferring phenotypic resistance from genomic data alone. Similar caution is warranted for *optrA*-associated linezolid resistance, as recent genomic studies have shown that *optrA* may occur in different chromosomal or plasmid contexts and may be embedded within broader MDR regions (26, 27). Overall, these findings support the complementary use of phenotypic antimicrobial susceptibility testing and WGS, rather than relying on either approach alone.

The 25 genome sequenced isolates displayed both diversity and localized clustering. The sequence-type distribution among the characterized isolates in our study indicated a more prominent expansion of ST16 among *E. faecalis* across several shelters of dogs and cats in the UAE. The dominance of ST16 among the genome-sequenced *E. faecalis* isolates is notable because this sequence type has been reported across clinical, environmental, and animal-associated compartments. Earlier comparative work on *E. faecalis* population structure reported ST16 as the only sequence type shared between human and poultry isolates, suggesting that this lineage may occur across host-associated niches relevant to One Health surveillance (28). In a clinical study from China, ST16 was one of the dominant *E. faecalis* sequence types among 265 isolates and was associated with strong or moderate biofilm formation, indicating traits that may support persistence in host-associated environments (29). The relevance of ST16 also extends to environmental reservoirs, as a linezolid-resistant *E. faecalis* ST16 isolate carrying *optrA* on a Tn6674-like element was recovered from surface water in Switzerland, with *optrA* linked to *fexA* and additional resistance determinants (30). In the present study, the detection of ST16 across multiple shelter-associated *E. faecalis* isolates, including the dog-shelter isolate Sfc9 carrying *optrA* in a predicted plasmidic context, adds a cautious One Health dimension to the genomic findings. Although these data do not establish transmission between animals, humans, or the environment, they indicate that shelter dogs may carry *E. faecalis* lineages and mobile resistance determinants that have also been documented in other One Health-relevant compartments. This supports the inclusion of shelter companion animals in genomic antimicrobial resistance surveillance, particularly when linezolid resistance determinants such as *optrA* are detected in predicted plasmid-associated or otherwise potentially mobile genetic contexts.

Virulence-associated gene profiling showed a species-structured pattern. The *E. faecium* isolates displayed adhesin-associated genes, whereas *E. faecalis* carried a broader set of functional categories, genes correlated with endocarditis- and biofilm-associated components, and the putative exoenzyme-associated determinants. This is consistent with previous observations that virulence determinants are often more common in *E. faecalis* than *E. faecium* (19). In Italy, a study found the gelatinase gene *gelE* in 62.6% of pet isolates, although only 26.1% produced gelatinase (20), illustrating that gene detection alone does not necessarily confirm expression. Therefore, the virulence gene patterns identified here should be interpreted as markers of potential colonization, adherence, or fitness traits, rather than direct evidence of pathogenicity.

This study has limitations that should be considered when interpreting the findings. Shelter participation was voluntary, and sampling was targeted rather than probability-based; therefore, the results provide baseline surveillance data but should not be extrapolated as national prevalence estimates. Additionally, the cross-sectional design did not allow assessment of persistence over time, and the absence of environmental, staff, and adopter samples limited source attribution and inference about animal–human exchange.

## Conclusion

Shelter dogs and cats in the UAE carried *E. faecalis* and *E. faecium* with phenotypic AMR and diverse genomic resistance determinants. Although vancomycin resistance was not detected, the incidental finding of linezolid resistance, along with detection of *optrA* on predicted plasmid associated contigs in *E. faecalis*, highlights the importance of including shelter companion animals in One Health AMR surveillance. Combining phenotypic susceptibility testing with WGS strengthened detection of resistance determinants, population structure, and virulence-associated traits, and provides a baseline model for future surveillance in the UAE and wider Middle East. Continued monitoring should include broader shelter sampling, environmental interfaces, longitudinal follow-up, and genomic approaches capable of resolving plasmid-mediated resistance.

## Data Availability

The datasets presented in this study can be found in online repositories. The names of the repository/repositories and accession number(s) can be found below: https://www.ncbi.nlm.nih.gov/, BioProject ID PRJNA1477438.
