# Supplementary material for: Shelter dogs and cats as a one health interface for phenotypic and genomic surveillance of antimicrobial-resistant Enterococcus Spp. in the United Arab Emirates
Source: Front Vet Sci. 2026 Jul 21;13:1906971. doi: 10.3389/fvets.2026.1906971 (PMC13435396; doi:10.3389/fvets.2026.1906971)
Supplement: Supplementary file 1 [file Image_1.pdf]

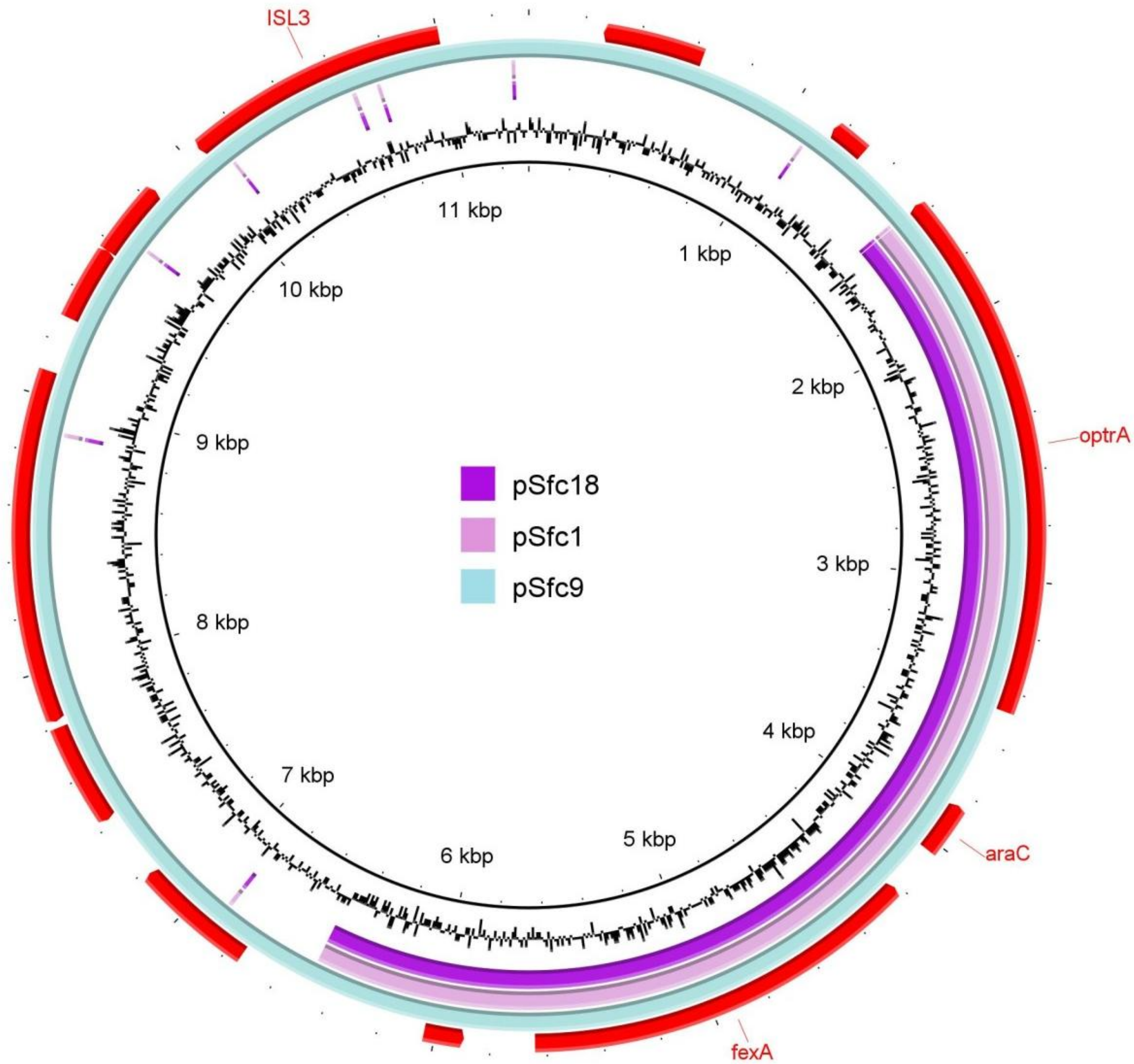

Supplementary Figure 1. Predicted plasmid context of *optrA*- and *fexA*-carrying *Enterococcus faecalis* isolates. Circular comparison map of three reconstructed plasmid sequences from *E. faecalis* isolates pSfc18, pSfc1, and pSfc9. Concentric coloured rings represent individual plasmid sequences aligned by sequence homology, with pSfc9 shown as the reference scaffold in light blue. The innermost track shows nucleotide sequence similarity across the aligned plasmid region, and plasmid coordinates are indicated in kilobase pairs (kbp). Annotated genomic features are shown in red, including the insertion sequence *ISL3* and resistance-associated loci *optrA* [oxazolidinone and phenicol resistance] and *fexA* [phenicol resistance], together with *araC*, a transcriptional regulator. Purple dashed marks indicate conserved homologous regions shared among the plasmid sequences. The co-localization of *optrA* and *fexA* on the same predicted plasmid context suggests linkage of oxazolidinone and phenicol resistance determinants within a shared mobile genetic region. Plasmid reconstruction and mobility/context prediction were performed using MOB-suite and gplasCC.
